# Haemodynamic monitoring during noncardiac surgery: past, present, and future

**DOI:** 10.1007/s10877-024-01161-2

**Published:** 2024-04-30

**Authors:** Karim Kouz, Robert Thiele, Frederic Michard, Bernd Saugel

**Affiliations:** 1 Department of Anesthesiology, Center of Anesthesiology and Intensive Care Medicine, University Medical Center Hamburg-Eppendorf, Martinistrasse 52, Hamburg, 20246 Germany; 2 Outcomes Research Consortium, Cleveland, OH USA; 3 Department of Anesthesiology, University of Virginia, Charlottesville, VA USA; 4 MiCo, Vallamand, Switzerland

**Keywords:** Anaesthesia, Blood pressure, Cardiac output, Haemodynamic monitoring, Heart rate

## Abstract

During surgery, various haemodynamic variables are monitored and optimised to maintain organ perfusion pressure and oxygen delivery – and to eventually improve outcomes. Important haemodynamic variables that provide an understanding of most pathophysiologic haemodynamic conditions during surgery include heart rate, arterial pressure, central venous pressure, pulse pressure variation/stroke volume variation, stroke volume, and cardiac output. A basic physiologic and pathophysiologic understanding of these haemodynamic variables and the corresponding monitoring methods is essential. We therefore revisit the pathophysiologic rationale for intraoperative monitoring of haemodynamic variables, describe the history, current use, and future technological developments of monitoring methods, and finally briefly summarise the evidence that haemodynamic management can improve patient-centred outcomes.

## Introduction

**Fig. 1:**
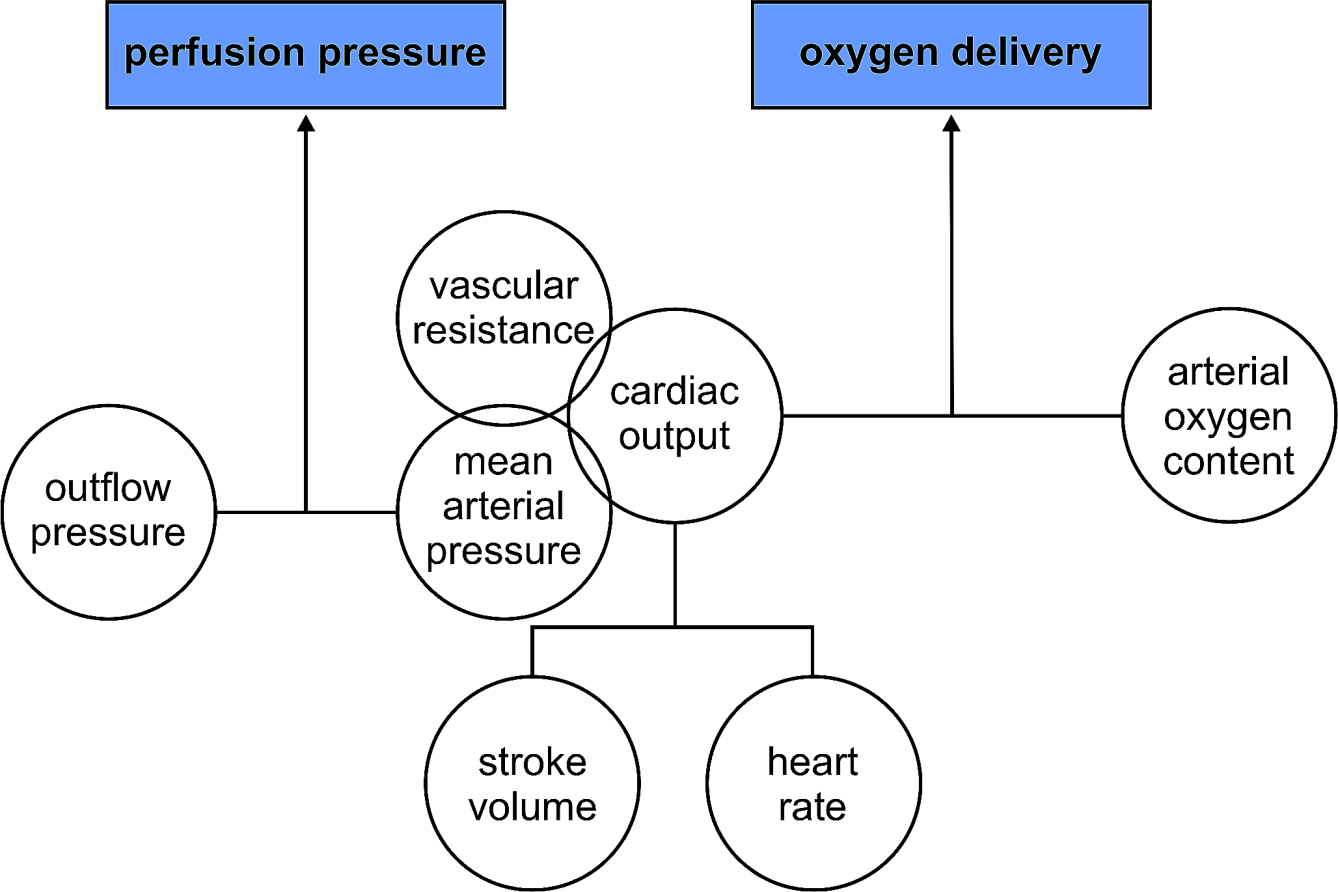
Haemodynamic variables determining perfusion pressure and oxygen delivery

Haemodynamic monitoring is the serial or continuous measurement of haemodynamic variables. Guiding therapeutic interventions based on haemodynamic monitoring is referred to as haemodynamic management. During surgery, the core objectives of haemodynamic monitoring and management are to ensure patient safety and to maintain organ perfusion pressure and oxygen delivery. Both adequate perfusion pressure and oxygen delivery are essential to maintain cellular metabolism of vital organs (Fig. 1) [1, 2]. The overarching goal of intraoperative haemodynamic monitoring and management is to maintain organ function and improve patient outcomes.

In this narrative review, we revisit the pathophysiologic rationale for intraoperative monitoring of haemodynamic variables. We further describe the history, current use, and future technological developments of monitoring methods. Finally, we briefly summarise the evidence that haemodynamic management can improve patient-centred outcomes.

### Heart rhythm and heart rate

Heart rate, together with stroke volume, is a main determinant of cardiac output (CO). Heart rate regulation is complex and includes neural und humoral control systems for short- and long-term adaptation of heart rate to metabolic needs. The normal heart rhythm is sinus rhythm, and the normal resting heart rate in adults is 60–100 beats per minute. Intraoperative heart rhythm and heart rate monitoring allows identifying cardiac arrythmias and abnormal high or low heart rates. Heart rhythm and heart rate monitoring are essential to ensure patient safety during surgery.

First attempts to monitor intraoperative heart rhythm and heart rate date back to 1896 when heart rate was assessed using a stethoscope to understand the effects of chloroform on cardiac physiology [3]. Later, this method was proposed to continuously monitor heart rhythm and rate [4]. In 1918, electrocardiography was first used to monitor intraoperative heart rate [5]. Four years later, a prospective study used electrocardiography to investigate the effects of anaesthesia and surgery on heart rhythm and rate [6]. In 1952, Himmelstein and Scheiner used an instrument called cardiotachoscope to continuously display the electrocardiogram and heart rate on a cathode ray screen [7]. Around 20 years later, intraoperative electrocardiography was proposed to detect acute myocardial ischemia [8].

Today, intraoperative heart rhythm and rate monitoring with electrocardiography is mandated by the European recommendations for standards of monitoring during anaesthesia and recovery [9] and the American Society of Anesthesiologists Standards for Basic Anesthetic Monitoring [10]. In the operating room, electrocardiography systems with three and five electrodes are most commonly used. The limb leads are typically placed on the shoulders, and the placement of the single precordial lead is variable and depends on the surgical procedure. The V_5_ lead is most sensitive to ST-segment changes, capturing 75% of events; V_4_ captures about 60% of ST-segment changes; the other precordial locations are significantly less sensitive [11]. Most electrocardiography systems use computerised ST-segment algorithms that compare the ST-segment and the iso-electric point from the PR-interval [12]. In addition to electrocardiography, photoplethysmography and intraarterial blood pressure waveforms can be used to derive pulse rate that equals heart rate when the patient has no pulse deficit.

Heart rates during surgery with general anaesthesia are usually lower than heart rates during physiologic sleep [13]. Patients on chronic beta blocker therapy are especially prone to develop intraoperative bradycardia [14]. Although intraoperative bradycardia can cause a decrease in CO and hypotension, it remains unknown what constitutes physiologically important intraoperative bradycardia [15]. Whether intraoperative bradycardia is related to organ injury is scarcely investigated. However, intraoperative bradycardia should presumably be treated when it is accompanied by profound hypotension or low CO [16, 17].

Intraoperative tachycardia is also common and can indicate hypovolaemia, inadequate depth of anaesthesia, or insufficient analgesia. A single-centre cohort study [18] and a secondary analysis [19] of the VISION study [20] suggest that intraoperative heart rates above 100 beats per minute are associated with myocardial injury, myocardial infarction, and death in patients having noncardiac surgery. In contrast, another single-centre cohort study in noncardiac surgery patients found no association between intraoperative heart rates above 80, 90, and 100 beats per minute and a composite outcome of myocardial injury and death [21].

In post-cardiac surgery patients with temporary epicardial pacing, individualised heart rate optimisation can help increase CO [22]. However, during noncardiac surgery, heart rate is rarely directly targeted and modified. There are thus no studies on the effect of intraoperative targeted heart rate management and outcomes. Beta blockers – such as metoprolol – may prevent intraoperative tachycardia and decrease the incidence of perioperative myocardial infarction but extended-release metoprolol is associated with perioperative hypotension and increased postoperative mortality in noncardiac surgery patients [23].

### Arterial pressure

Arterial pressure results from the interaction between CO and systemic vascular tone – and is characterised by three components, namely, systolic, mean, and diastolic arterial pressure. Mean arterial pressure – the mean pressure over the cardiac cycle – is the inflow pressure for most organs, while the outflow pressure is the higher of either central venous pressure (CVP) or extravascular pressure in a specific tissue, organ, or compartment [24, 25]. Systolic arterial pressure is determined by left ventricular stroke volume, vascular compliance, backward reflected waves, and pulse amplification and reflects left ventricular afterload [24, 25]. Diastolic arterial pressure is primarily determined by systemic vascular tone [24, 25]. The difference between systolic and diastolic arterial pressure is called pulse pressure and closely reflects stroke volume.

The beginning of intermittent oscillometric monitoring dates back to the beginning of the 19th century when sphygmomanometers were used to measure arterial pressure during surgery [26, 27]. However, it took around 50 years until arterial pressure gained attention in perioperative medicine. In 1951, shortly after introducing oscillometry during anaesthesia [28], systolic arterial pressures of 30 mmHg were considered safe in young and healthy patients [29]. First attempts to measure arterial pressure with a plastic catheter inserted into a peripheral artery in humans date back to 1949 [30]. However, at that time, this method was considered to be “unsuitable for routine use during anaesthesia” because it “requires the introduction of a thin plastic catheter into a peripheral artery” [31]. In 1986, the American Society of Anesthesiologists formulated that arterial pressure monitoring is mandatory during anaesthesia [32]. Today, the American Society of Anesthesiologists Standards for Basic Anesthetic Monitoring mandate the measurement of arterial pressure at least every five minutes [10].

Currently, two methods are routinely used to monitor arterial pressure during surgery: intermittent oscillometric monitoring with an upper-arm cuff and continuous intraarterial monitoring with an arterial catheter [33–35]. Continuous noninvasive monitoring with finger-cuffs is also available – but not yet implemented for routine use [33–35].

Automated oscillometry is noninvasive, easy to use, and comparatively cheap. An obvious limitation is that oscillometry provides arterial pressures only intermittently. Furthermore, the measurement performance of oscillometry is highly dependent on the measurement site [36], appropriate cuff size [37], and cuff position [38]. Notably, oscillometry overestimates low and underestimates high arterial pressures – and may thus miss hypotension and hypertension [39–41].

**Fig. 2:**
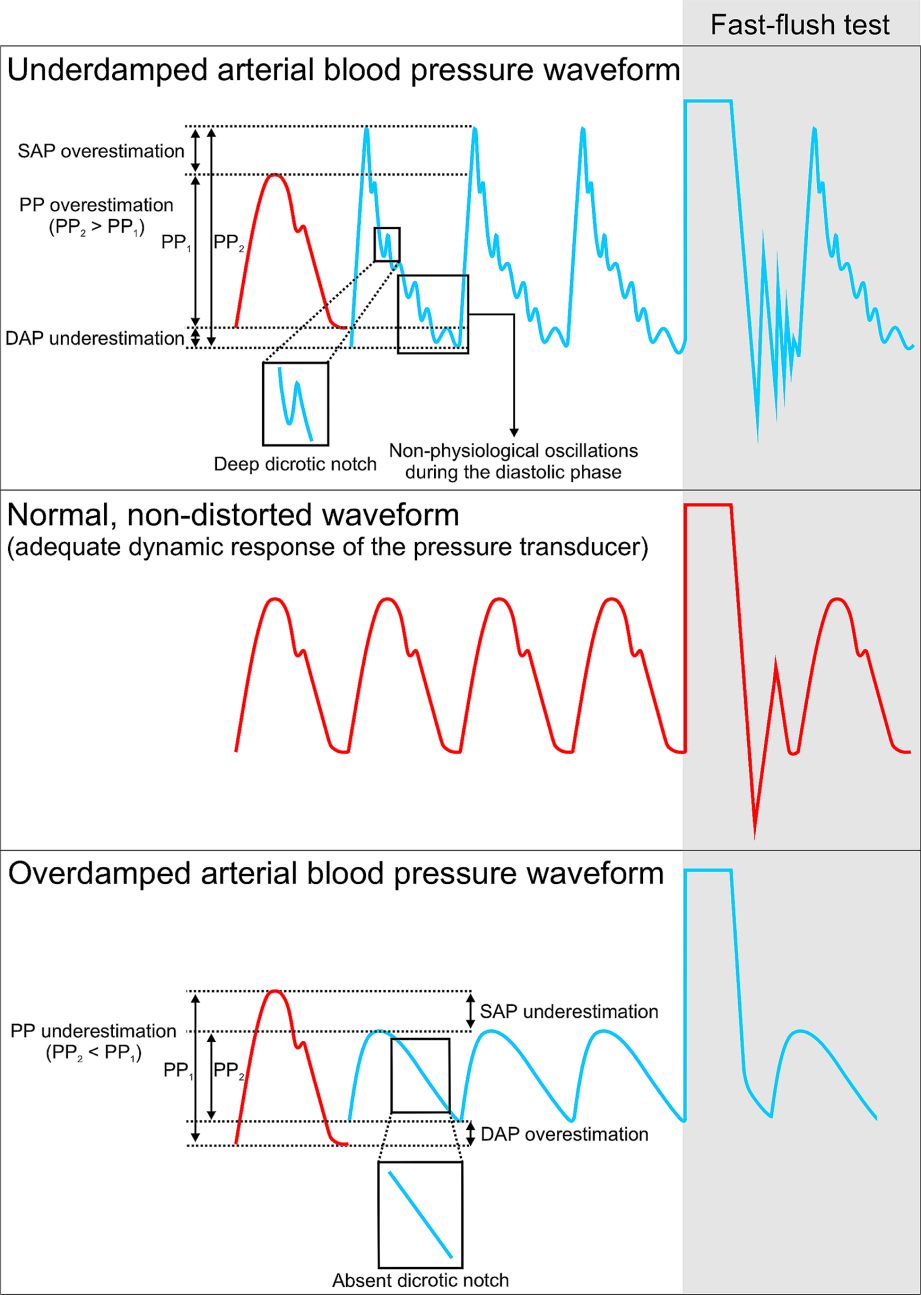
Dynamic response of the arterial pressure measurement system. This figure illustrates an adequately damped arterial pressure waveform and characteristic changes of the arterial pressure waveform when underdamping and overdamping is present. The red arterial pressure waveform represents a “normal,” non-distorted waveform with a normal fast-flush test, whereas the blue arterial pressure waveforms represent an underdamped (upper part of the figure) or overdamped (lower part of the figure) arterial pressure waveform. PP, pulse pressure; SAP, systolic arterial pressure; DAP, diastolic arterial pressure. “Under- and overdamping of the arterial blood pressure waveform and fast-flush test” by Saugel et al. [42] is licensed under CC BY 4.0

Intraarterial monitoring with an arterial catheter is the clinical reference method to continuously measure arterial pressure [42]. Serious complications caused by arterial catheters – such as ischemia or major bleeding – are very rare [43]. While intraarterial monitoring is more accurate than oscillometry, it does require that the measurement system is properly levelled or zeroed and damped to avoid overdamping or underdamping (Fig. 2) [35, 42]. Intraarterial monitoring may help reduce hypotension during and after anaesthetic induction as well as during surgery compared to intermittent oscillometric monitoring [44, 45].

An alternative to intermittent noninvasive oscillometric and continuous intraarterial monitoring is continuous noninvasive arterial pressure monitoring using the finger-cuff-based vascular-unloading technique [33]. Validation studies investigating the measurement performance of the vascular-unloading technique *versus* intraarterial monitoring revealed heterogeneous results [46]. Several studies demonstrated interchangeability between arterial pressure measurements with the vascular-unloading technique and intraarterial measurements, but only one third of studies reported accuracy and precision meeting current international standards [46]. Importantly, the vascular-unloading technique provides arterial pressures continuously and its measurement performance seems to be at least as good as that of intermittent oscillometry [36, 47]. However, in patients with circulatory shock or high-dose vasopressor therapy the vascular-unloading technique becomes unreliable because of impaired finger perfusion [48, 49]. While the accuracy of the vascular-unloading technique is yet to be fully established, continuous monitoring using the vascular-unloading technique reduces the incidence of post-induction and intraoperative hypotension when compared to intermittent oscillometric monitoring [50, 51]. Miniaturised wireless systems with sensors integrated in a finger-ring are currently being developed [52].

The pulse decomposition method allows continuously reconstructing arterial pressure waveforms from a finger-cuff and a piezo electric sensor [53]. First studies suggest that this new noninvasive method meets current international standards for arterial pressure monitoring both in surgical [54] and critically ill patients [55].

The hydraulic coupling method has been proposed to noninvasively measure arterial pressure [56]. The main advantage of this technology is that it increases the signal-to-noise ratio compared to conventional oscillometry by using silicon-oil instead of air to transmit oscillations [56, 57]. A first validation study performed by the developers reported good agreement with intraarterial measurements from femoral arterial catheters [56]. In addition, the hydraulic coupling method allows reconstructing arterial pressure waveforms [58].

Artificial intelligence can be used to analyse the arterial pressure waveform to predict hypotension or identify underlying causes of hypotension. One of the first attempts to use artificial intelligence to predict hypotension by analysing arterial pressure waveform features is the hypotension prediction index software (HPI-software) (Edwards Lifesciences, Irvine, CA, USA) [59]. A registry study suggests that using HPI-software monitoring may help clinicians reduce intraoperative hypotension during noncardiac surgery [60, 61]. However, trials investigating the effect of HPI-software monitoring on intraoperative hypotension revealed contradictory results: while a small trial suggested that HPI-software monitoring helps reduce hypotension [62], a larger trial did not [63]. There are ongoing scientific controversies around HPI-software validation [64] and on whether HPI values just reflect mean arterial pressure values or provide predictive capabilities beyond changes in mean arterial pressure per se [65, 66].

Artificial intelligence can also help identify root causes of hypotension. In patients having major abdominal surgery, artificial intelligence was used to identify endotypes of intraoperative hypotension [15]. During episodes of hypotension, an unsupervised machine learning algorithm grouped measurements of stroke volume index, heart rate, cardiac index, systemic vascular resistance index, and pulse pressure variation (PPV) into hypotension endotypes, namely, myocardial depression, bradycardia, vasodilation, hypovolaemia, and mixed endotype [15]. It remains to be determined if considering hypotension endotypes helps treat hypotension causally and improve outcomes.

Although underlying causes of intraoperative hypotension are well described, individual hypotension harm thresholds remain largely unknown. On a population basis, intraoperative mean arterial pressures below 60–70 mmHg are associated with organ injury [67–73]. Organ injury is a function of hypotension severity and duration. Harm from hypotension accrues at profoundly low pressures rather than from long exposure to moderately low pressures [74]. While the association between intraoperative hypotension and organ injury is well established, it remains largely unknown whether the association between intraoperative hypotension and complications observed in registry studies is indeed causal – and thus amenable to interventions. Additionally, although intraoperative hypotension at some level causes organ injury, harm thresholds for individual patients also remain unclear [75–78]. Universally targeting mean arterial pressures higher than 60 mmHg during surgery does not reduce postoperative complications in noncardiac surgery patients [77, 78]. In contrast, individualising intraoperative arterial pressure targets based on preoperative resting arterial pressures reduced postoperative complications compared to routine arterial pressure management in a multicentre trial of 298 noncardiac surgery patients [75]. Ongoing trials will provide more evidence on the effect of using fixed (NCT04884802) or individualised [79] intraoperative arterial pressure targets on outcomes of high-risk noncardiac surgery patients.

### Pulse pressure variation and stroke volume variation

**Fig. 3:**
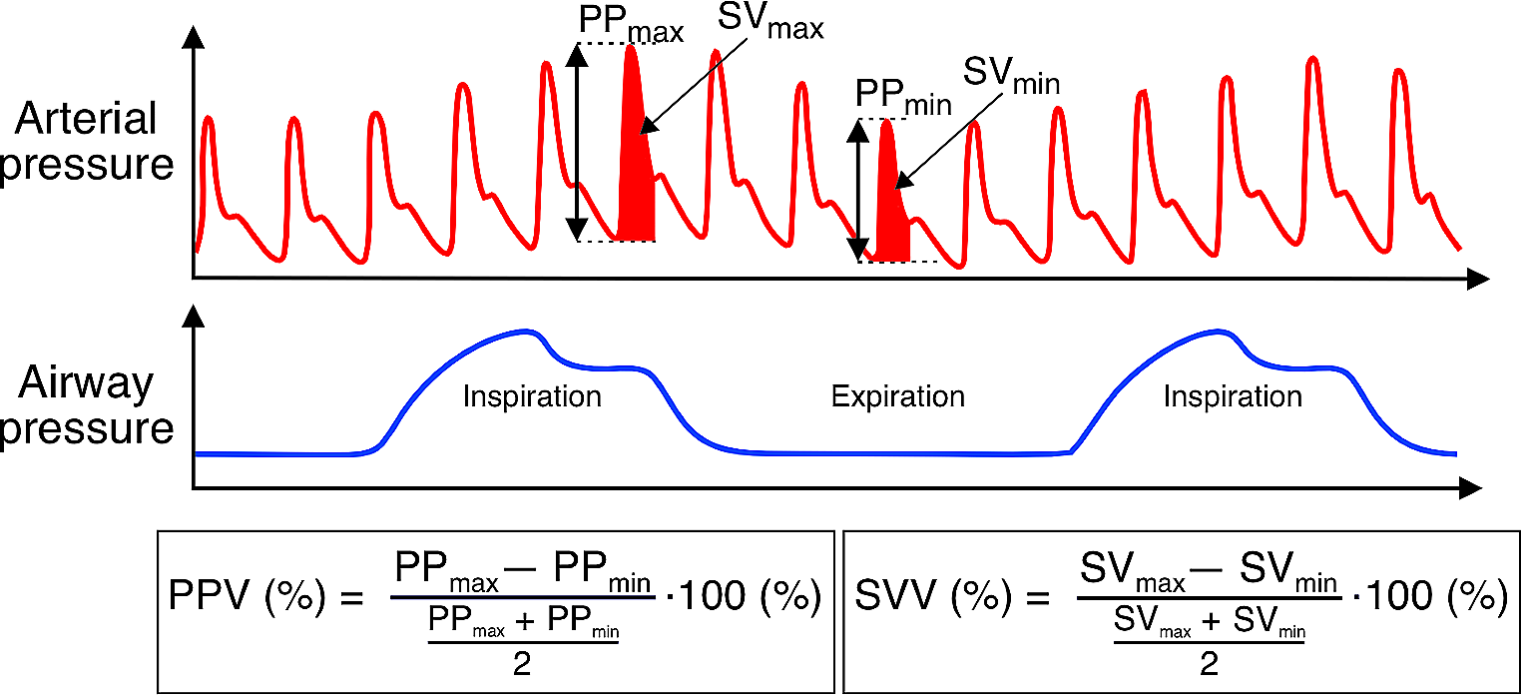
Cyclic changes in arterial pressure during mechanical ventilation and calculation of the dynamic cardiac preload variables pulse pressure variation and stroke volume variation. PP, pulse pressure; PPV: pulse pressure variation; SV, stroke volume; SVV, stroke volume variation

PPV and stroke volume variation (SVV) are dynamic variables that can be used to predict fluid responsiveness in mechanically ventilated patients (Fig. 3) [80, 81]. PPV and SVV primarily reflect cyclic changes in left ventricular stroke volume that are caused by positive pressure ventilation in mechanically ventilated patients [82].

Cyclic changes in venous return and aortic blood flow in mechanically ventilated patients were first reported in 1966 [83]. First attempts to quantify these changes date back to 1978 [84]. Research on the relation between systolic pressure variation and blood volume [85, 86] finally lead to the use of PPV [87] and SVV [88] to predict fluid responsiveness. Today, many regular bedside monitors automatically provide PPV. In contrast, SVV naturally requires the estimation of stroke volume with advanced haemodynamic monitoring systems. PPV also can be calculated based on noninvasively obtained continuous arterial pressure waveforms [58, 89]. Recently, it has been shown that the hydraulic coupling method [56] allows the reconstruction of the arterial pressure waveform and calculation of PPV [58].

PPV and SVV continuously provide information on fluid responsiveness and predict fluid responsiveness more accurately than static preload variables – e.g., CVP [90]. Additionally, predicting fluid responsiveness with PPV and SVV does not require fluid administration (like when performing fluid challenges [91]) or patient positioning (like when performing passive leg raising tests [92] that are usually impossible during surgery). However, certain clinical situations preclude the use of dynamic cardiac preload variables – including cardiac arrhythmias, tidal volumes of less than 7–8 ml/kg, and high intraabdominal pressure (e.g., during laparoscopic surgery) [93, 94]. In patients with low tidal volume ventilation, a tidal volume challenge may help increase the predictive value of PPV and SVV to reliably predict fluid responsiveness [95]. Whenever PPV or SVV cannot be used, other tests including fluid challenges [91], passive leg raising tests [92], and end-expiratory occlusion tests [96] may be used to assess fluid responsiveness. For both PPV and SVV thresholds of 11% have been suggested to predict fluid responsiveness [97]. However, PPV values in a ‘grey zone’ between 9 and 13% [98] are inconclusive regarding fluid responsiveness. PPV and SVV are frequently used to titrate fluid administration within perioperative goal-directed haemodynamic therapy protocols and may help reduce net fluid administration and postoperative complications [99, 100] – mainly when fluid management based on dynamic cardiac preload variables is combined with blood flow optimisation [101].

### Central venous pressure

CVP is the venous pressure in the superior vena cava near the right atrium and thus an estimate of right atrial pressure.

The first catheterisation of a central vein was performed in 1733 with a glass tube introduced in the jugular vein of a horse to measure CVP [102]. About two centuries later, Werner Frossman inserted a urinary catheter into his arm vein up to his right heart [103]. Thereafter, it again took some years before clinicians started measuring CVP to guide haemodynamic therapy [104].

**Fig. 4:**
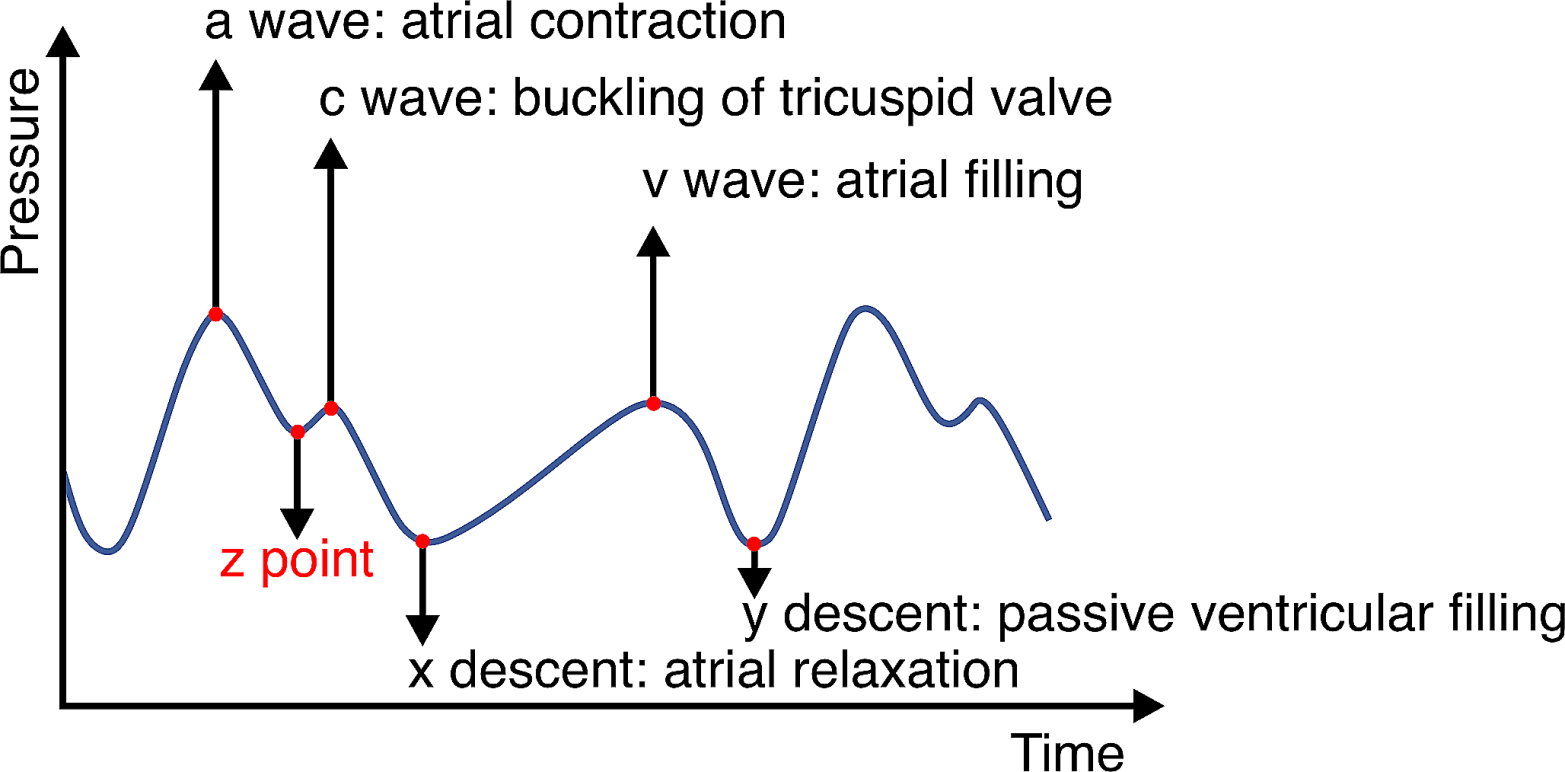
Central venous pressure curve

CVP can be measured in patients with a central venous catheter or a dedicated pulmonary artery catheter with a right atrial port. CVP should ideally be measured at end-expiration at the base of the c wave (so called z-point) because this point reflects the final pressure in the ventricle before onset of systole (Fig. 4) [105]. However, the c wave is not always easy to identify and in practice the mean CVP or the pressure at the base of the a wave is considered. CVP values and waveforms are influenced by numerous factors, including blood volume, cardiac function and pathologies, intrathoracic pressure, and venous compliance [105]. Although CVP is not a good marker of fluid responsiveness [90, 106] or volume status [107], it may be useful as a marker of right heart function. In addition to the absolute value of the CVP, the CVP waveform – which consists of a, c, and v waves, as well as x and y descents – may help identify pathophysiologic situations. For example, a loss of the a wave occurs in atrial fibrillation, and high v waves occur in patients with tricuspid regurgitation. Because absolute mean CVP values are normally close to zero in spontaneously breathing patients, correct levelling or zeroing of the measurement system is crucial [108]. Furthermore, considering transmural pressure is essential when interpreting CVP because CVP changes during the respiratory cycle and is affected by the positive end-expiratory pressure during mechanical ventilation.

Recent advances in CVP monitoring include noninvasive estimation of CVP using jugular near infrared spectroscopic sensors [109, 110]. However, as of today the number of validation studies investigating this method is very limited and the clinical usefulness remains unknown [109, 110]. CVP may be estimated without a central venous catheter from peripheral venous pressure as a surrogate for CVP [111–114].

During intraoperative haemodynamic management, CVP should not be used as a target variable. CVP should rather be considered a safety variable – with high (and especially rapidly increasing) CVP indicating haemodynamic problems such as acute right heart failure or right ventricular out-flow tract obstruction. Considering CVP changes in the context of other haemodynamic variables may help understand haemodynamic alterations.

### Cardiac output and stroke volume

CO is the product of stroke volume and heart rate. CO – together with arterial oxygen content – determines oxygen delivery. Tissue hypoperfusion may result in organ injury [2]. The rationale to monitor CO during surgery thus is to avoid organ injury by maintaining oxygen delivery. Additionally, knowing stroke volume and CO helps understand the underlying mechanisms of haemodynamic instability that may include a decrease in blood flow or a decrease in vascular tone. Current guidelines suggest that CO or stroke volume monitoring may be considered in patients with a high risk for complications [115].

First attempts to compute CO in animals date back to 1870, when Adolf Fick described the Fick’s principle. Fick’s original principle was based on the extraction of oxygen through the systemic circulation. Later on, the Fick principle was adapted and used for the development of indicator dilution methods in 1897 [116] and thermodilution methods in 1954 [117]. In parallel, Otto Frank started the first attempts to estimate stroke volume and CO using pulse wave analysis based on the Windkessel model – a model that assumes that at a steady haemodynamic state, the amount of blood entering a blood vessel is equal to the amount of blood leaving the vessel during the cardiac cycle [118]. Based on the initial research of Fick and Frank, two CO monitoring methods that are still routinely used today were developed: thermodilution [119] and pulse wave analysis [120–122].

**Fig. 5:**
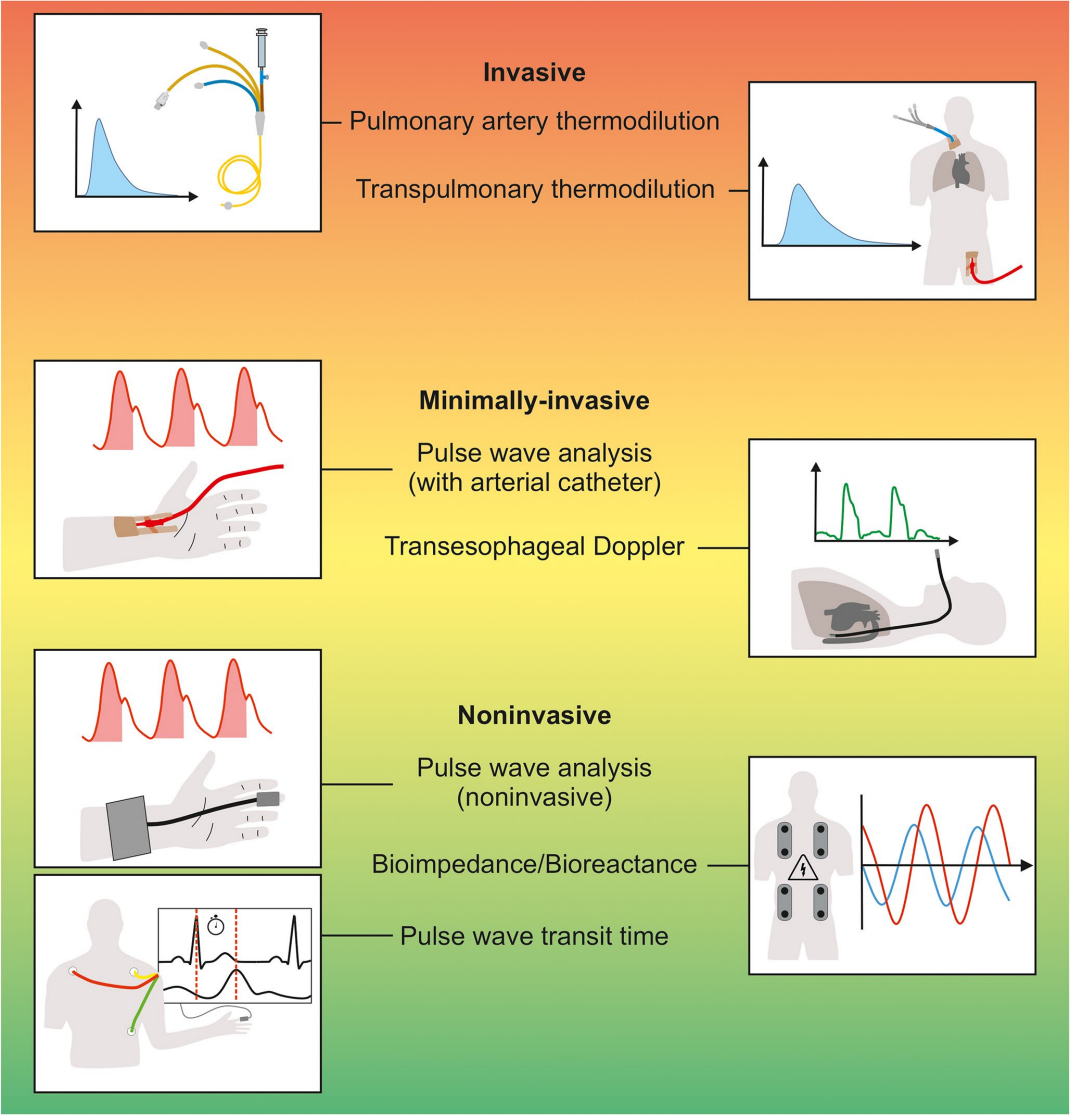
Cardiac output monitoring methods classified according to their invasiveness into invasive, minimally-invasive, and noninvasive methods

There are numerous methods to measure stroke volume and CO in patients having surgery – including pulmonary artery or transpulmonary thermodilution, pulse wave analysis, oesophageal Doppler, and bioreactance/bioimpedance techniques (Fig. 5) [123–126]. Thermodilution methods are considered clinical reference methods to measure CO [119], but – due to their invasiveness – they are rarely used in patients having noncardiac surgery. Thermodilution methods are thus reserved for special indications including liver transplant and cardiac surgery [124]. Pulse wave analysis is commonly used to measure CO during surgery [127].

**Fig. 6:**
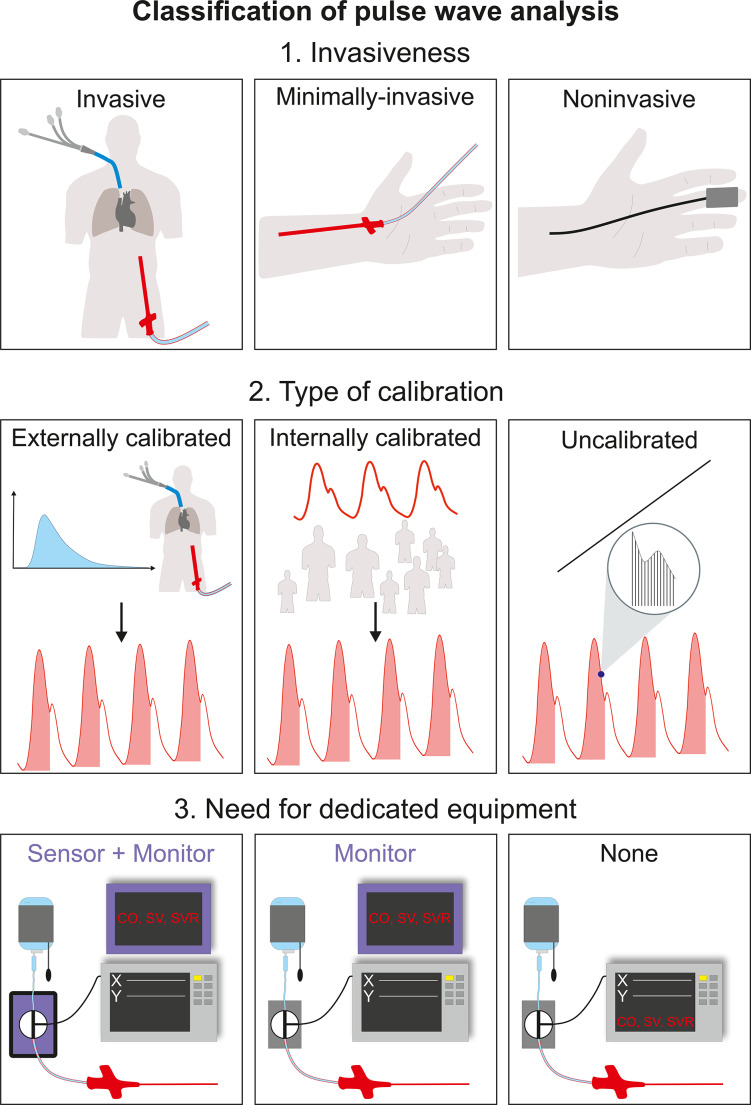
Classification of pulse wave analysis according to invasiveness, type of calibration, and need for dedicated equipment

Pulse wave analysis algorithms continuously analyse the arterial pressure waveform to estimate stroke volume and CO [120–122]. Pulse wave analysis systems can be classified as invasive/minimally-invasive or noninvasive – depending on whether the arterial pressure waveform is measured with an arterial catheter or a noninvasive sensor (Fig. 6) [120–122]. The systems can additionally be classified considering the type of calibration. Externally calibrated systems are calibrated using an external measurement technique that usually is an indicator dilution method [120–122]. Internally calibrated systems use biometric, demographic, and haemodynamic data to calibrate pulse wave analysis-derived CO values [120–122]. Uncalibrated systems rely on special algorithms such as the pressure recording analytical method allowing beat-to-beat impedance estimations and further calculation of haemodynamic variables [120–122]. An advantage of pulse wave analysis is the continuous estimation of stroke volume. However, the measurement performance of pulse wave analysis can be impaired when vascular tone is substantially altered or rapidly changing [128]. For pulse wave analysis being able to accurately estimate stroke volume, the analysed arterial pressure waveform needs to be correctly damped, i.e. the dynamic response of the measurement system needs to be adequate [42, 129]. Using mechanical or electronic filters to identify and correct abnormal waveform damping can help improve pulse wave analysis-derived stroke volume and CO measurements [129].

With the oesophageal Doppler method stroke volume is estimated based on blood flow velocity in the descending aorta and the aortic cross-sectional area (assuming that blood flow distribution between the upper and lower parts of the arterial system is constant) [130]. The oesophageal Doppler method allows continuous beat-to-beat stroke volume estimation independent from changes in vascular tone, but the measurement performance substantially depends on the correct estimation of the diameter of the aorta used to calculate the aortic cross-sectional area [131]. Additionally, the oesophageal Doppler probe needs to be frequently repositioned and thus requires user attention and is operator dependent.

Thoracic bioimpedance/bioreactance are noninvasive methods to estimate stroke volume and CO by measuring the frequency modulation when an oscillating voltage is applied across the thorax [126, 132–134]. In short, these methods estimate the volume of electrically conducting blood moving in and out of the chest as a surrogate for stroke volume [126, 132–134]. Bioimpedance/bioreactance measurements can be disturbed by motion, electrical interference, arrhythmias, pleural effusion, pulmonary oedema, and mechanical ventilation [126, 132–134].

CO is determined by metabolic needs [135]. Therefore, there is no “normal CO”. Resting CO varies substantially among individuals – but generally decreases with age [136]. Although arterial pressure and CO are physiologically coupled, there is no clinically meaningful correlation between arterial pressure and CO in patients having surgery [137]. Perioperative CO-guided management – often subsumed under the umbrella term “goal-directed haemodynamic therapy” [138] – was proposed in the 1970s by William C. Shoemaker [139]. Since then, numerous – mainly small and fragile – trials investigated the effect of different CO-guided management strategies on patient outcome. While CO targets and therapeutic interventions substantially differ among trials [140, 141], cumulative evidence suggests that CO-guided management may help reduce postoperative complications and even mortality [99, 142–146]. However, in the largest trial so far, the OPTIMISE II trial, maximising stroke volume using fluids and dobutamine did not reduce the incidence of postoperative infectious complications or any other complication (OPTIMISE II trial [147]; Presented at EBPOM World Congress of Prehabilitation Medicine 2023 in London on July 6, 2023).

Recent developments in the field of intraoperative CO monitoring focus on accessibility and sustainability [148]. To be implemented in routine care, CO monitoring systems need to be accessible. Costs of haemodynamic monitoring equipment is still perceived as a major barrier to hospital adoption [149, 150]. Sustainability is an increasing concern in anaesthesiology [151]. Disposable-free monitoring solutions have the advantage to decrease plastic waste, carbon dioxide emission, and costs [148].

## Summary

During surgery, various haemodynamic variables are monitored and optimised to ensure patient safety, maintain organ perfusion pressure and oxygen delivery, and eventually avoid organ injury and improve patient outcomes. Important haemodynamic variables that provide an understanding of most pathophysiologic haemodynamic conditions during surgery include heart rate, arterial pressure, CVP, PPV, SVV, stroke volume, and CO. Future research should focus on the development of accessible and sustainable monitoring methods that reliably measure haemodynamic variables – preferably in a wireless, interconnected, and noninvasive manner.

## Data Availability

No datasets were generated or analysed during the current study.
